# Investing in health systems for universal health coverage in Africa

**DOI:** 10.1186/s12914-014-0028-5

**Published:** 2014-10-28

**Authors:** Luis Gomes Sambo, Joses Muthuri Kirigia

**Affiliations:** 1 World Health Organization, Regional Office for Africa, Brazzaville, Congo; 2 Research, Publications and Library Services Programme, Health Systems and Services Cluster, World Health Organization, Regional Office for Africa, Brazzaville, Congo

**Keywords:** Health systems, Health workforce, Health facilities, Health technologies, Health financing, Information, Leadership and governance, Health interventions, Universal health coverage, African region

## Abstract

**Background:**

This study focused on the 47 Member States of the World Health Organization (WHO) African Region. The specific objectives were to prepare a synthesis on the situation of health systems¿ components, to analyse the correlation between the interventions related to the health Millennium Development Goals (MDGs) and some health systems¿ components and to provide overview of four major thrusts for progress towards universal health coverage (UHC).

**Methods:**

The WHO health systems framework and the health-related MDGs were the frame of reference. The data for selected indicators were obtained from the WHO World Health Statistics 2014 and the Global Health Observatory.

**Results:**

African Region¿s average densities of physicians, nursing and midwifery personnel, dentistry personnel, pharmaceutical personnel, and psychiatrists of 2.6, 12, 0.5, 0.9 and 0.05 per 10 000 population were about five-fold, two-fold, five-fold, five-fold and six-fold lower than global averages.

Fifty-six percent of the reporting countries had fewer than 11 health posts per 100 000 population, 88% had fewer than 11 health centres per 100 000 population, 82% had fewer than one district hospital per 100 000 population, 74% had fewer than 0.2 provincial hospitals per 100 000 population, and 79% had fewer than 0.2 tertiary hospitals per 100 000 population.

Some 83% of the countries had less than one MRI per one million people and 95% had fewer than one radiotherapy unit per million population. Forty-six percent of the countries had not adopted the recommendation of the International Taskforce on Innovative Financing to spend at least US$ 44 per person per year on health. Some of these gaps in health system components were found to be correlated to coverage gaps in interventions for maternal health (MDG 5), child health (MDG 4) and HIV/AIDS, TB and malaria (MDG 6).

**Conclusions:**

Substantial gaps exist in health systems and access to MDG-related health interventions. It is imperative that countries adopt the 2014 Luanda Commitment on UHC in Africa as their long-term vision and back it with sound policies and plans with clearly engrained road maps for strengthening national health systems and addressing the social determinants of health.

## Background

The World Health Organization (WHO) defines health as a state of complete physical, mental and social well-being and not merely the absence of disease or infirmity [1]. In line with the United Nations Universal Declaration of Human Rights [2], the WHO constitution states that the enjoyment of the highest attainable standard of health is one of the fundamental rights of every human being without distinction of race, religion, political belief or economic or social condition. The United Nations Sustainable Development Solutions Network (UNSDSN) advocates for maximisation of health well-being for all ages through universal health coverage (UHC) and pro-health policies in all sectors [3].

The Fifty-eighth World Health Assembly defined UHC as access of all population to key promotive, preventive, curative, rehabilitative and palliative health interventions at an affordable cost, thereby achieving equity in access [4]. UNSDSN defines UHC as equitable access to affordable, accountable, appropriate health services of assured quality by all people, including promotive, preventive, curative, palliative and rehabilitative services [3]. Following a critical examination of whether the UHC goal would be consistent with the right to health, Ooms and colleagues [5] concluded that UHC, as proposed by the UNSDSN, could be called a practical expression of the right to health care.

The purposes of UHC are to meet population needs for quality health care, remove financial barriers to health care access, reduce incidence of catastrophic health expenditures, attain national and internationally agreed health goals, and ultimately contribute to poverty alleviation and development [6]. There is evidence that broad health coverage, facilitated by extended risk pooling and prepayment, generally leads to better access to necessary care and improved population health, particularly for poor people [7].

According to the UN Special Rapporteur, Paul Hunt, existence of an effective and integrated health system encompassing health care and the underlying determinants of health, responsive to national and local priorities and accessible to all is a prerequisite for attainment of the right to health [8]. Hunt advocates for systematic application of the right to health principles to the six WHO health system building blocks of service delivery, health workforce, information, financing, leadership/governance, and medical products, vaccines and technologies [9].

The 2009 Ouagadougou Declaration on Primary Care and Health Systems in Africa outlines the generic interventions that countries could implement to address the unrelenting regional challenges facing the health systems [10]. The Ouagadougou Declaration urges countries to align their national health policies with the requirements of the primary health care approach in order to achieve UHC. The framework for implementing the Ouagadougou Declaration proposes generic interventions that countries could adopt to strengthen health service delivery leadership and governance, human resources for health, health financing, health technologies, community ownership and participation, and partnerships for health development [11]. The 2014 Luanda Commitment on UHC in Africa reignited the flame for the need to implement the strategies called for by the Ouagadougou Declaration, since all 54 African Union Member States, 47 of which belong to the WHO African Region, pledged to put in place by 2025 the necessary structures and processes to ensure that they can attain UHC [12].

Concerned about the inadequate implementation of ministerial resolutions, the Luanda meeting also committed to the creation of an accountability mechanism to assess the implementation of commitments made by African ministers of health [12]. It is important to assess the situation of health systems in the African Region and to monitor over time their progress towards achieving the vision of UHC [13],[14].

A number of studies in the African Region have attempted to analyse the condition of individual components of health systems such as financing [15],[16], human resources [17], information and information systems [18], national research systems [19],[20], research ethics [21], and services [22]-[27], but no empirical study on leadership [28] and governance of health development [29] has been carried out. Also, there is a dearth of literature comprehensively synthesising the situation of health systems in the Region. The aim of this study is to contribute to the bridging of the information gap using the latest data available on health systems¿ components. The specific objectives were to prepare a synthesis on the current situation of health systems¿ components, analyse the correlation between health MDG-related interventions and some health systems¿ components, and to provide overview of four major thrusts towards UHC.

## Methods

### Overview of the WHO health system framework

**Figure 1:**
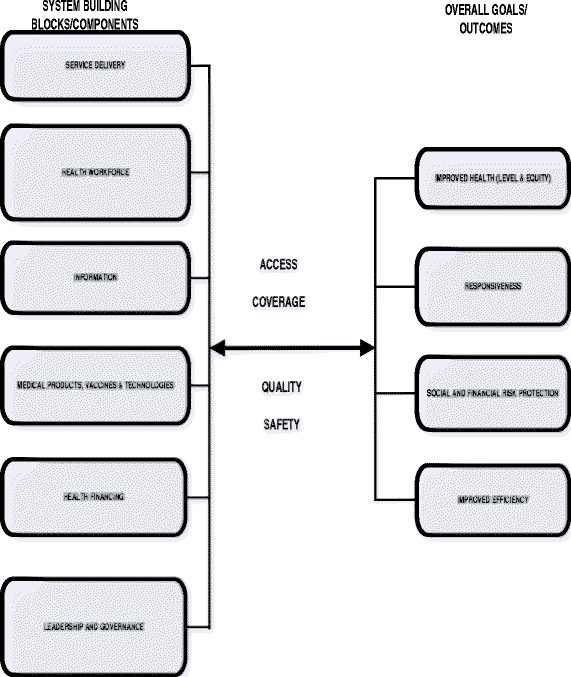
The WHO Health system framework.

Murray and Frenk [30] defined a health system as all organisations, people and actions whose primary intent is to promote, restore or maintain health. As shown in Figure 1, according to WHO a national health system has six core components [9]: health workforce, service delivery, health technologies (medical products, vaccines & technologies), financing, information, and leadership and governance.

Human resources for health refer to a health workforce that functions in ways that are responsive, fair and efficient to achieve the best health outcomes possible given the available resources.

Health facilities are meant to deliver effective, safe, quality personal and non-personal health care interventions to those in need of them [9]. Health care comprises a continuum of services, ranging from home-based, self-administered treatment to services provided at a health post or centre, or a district, provincial or highly specialised tertiary hospital.

A health post provides limited ambulatory and curative services and participates in some community development activities [31]. It is meant to serve a catchment population of 3000 to 5000. A health centre on the other hand provides a full range of health promotion and preventive services including maternal and child health services and ambulatory and curative health care, as well as serving as a support reference for health posts [31]. A typical health centre would serve a population of 10 000 [32].

As the first referral point, a district hospital ought to offer diagnostic, treatment, care, counselling and rehabilitation services. A district hospital would service a population of about 100 000 to 150 000 [33]. A provincial hospital is considered as a secondary level health facility highly differentiated by function, with five to 10 clinical specialties and a capacity of 200 to 800 beds [34]. Tertiary level hospitals have highly specialised staff, advanced technology, highly differentiated clinical functions such as health care services and teaching and research, and a capacity of 300 to 1500 beds [34].

Health technologies concern essential medical products, vaccines and medical devices, all which should be of assured quality, safety and efficacy and be cost-effective to use [9].

Financing refers to a system that mobilises adequate funds for health in ways that ensure access to needed services and protection from the financial catastrophe associated with health care spending.

The information component is about data production, analysis, reporting, dissemination and use of reliable and timely information on health determinants, health systems performance and health status of the population [9].

Leadership and governance component is concerned about ensuring that strategic policy frameworks exist and are combined with effective oversight, building of partnerships and application of appropriate regulations for policy and decision-making.

WHO [9] defines the overall health system goal as improvement of health and health equity in ways that are responsive, financially fair and make efficient use of available resources. A national health system uses human resources for health, health facilities, health technologies, health financing, information, and leadership and governance to produce and deliver health services such as the interventions associated with the health MDGs. Health system goal is attained through access to and ensuring coverage of quality, safe and effective health interventions or services.

### Analysis

#### Objective 1: Prepare a synthesis on the situation of health systems¿ components

We analysed data on the distribution of the key types of human resources for health, health facilities, health technologies, health financing, information, and leadership and governance. This entailed calculation of frequencies and descriptive statistics, as reported under ¿Synthesis of health system components¿ in the ¿Results and discussion¿ section.

#### Objective 2: Analyse the correlation between health MDG-related interventions and some health systems¿ components

This objective was addressed through analysing frequency distributions on coverage of public health interventions related to MDG 5 on improving maternal health, MDG 4 on reducing child mortality, MDG 6 on combating HIV/AIDS, malaria and other diseases, and MDG 8 on creating a global partnership for development. For MDG 8 we dealt with only Target 8.E, which reads ¿In cooperation with pharmaceutical companies, provide access to affordable essential drugs in developing countries¿. We used simple linear regression to estimate correlation coefficients (r) and the t-test of the null hypothesis (H_0_) that there was no relationship between pairs of the variables concerned. Details on the analysis are shown under ¿Progress towards universal coverage of MDG-related interventions¿ in the ¿Results and discussion¿ section.

#### Objective 3: Provide overview of four major thrusts towards universal health coverage

To address the gaps in providing access to interventions related to health MDGs, we interrogated the current issues and challenges to draw up the four thrusts that we propose as essential actions for the countries to advance towards UHC. The argument for these thrusts is presented under ¿Major thrusts towards universal health coverage¿ in the ¿Results and discussion¿ section.

### Data

All the 47 countries in the WHO African Region in 2014 were considered in this study. The variables analysed fell into eleven categories. The first category covered human resources for health, specifically physicians; nursing and midwifery, dentistry and pharmaceutical personnel; and psychiatrists [35],[36].

The second category dealt with health facilities, namely health posts and centres and district, regional and tertiary hospitals [36].

The third category, on health technologies, was concerned with densities of magnetic resonance imaging, radiotherapy and computed tomography units [36], median availability of selected generic medicines and vaccine coverage [35]. This category also includes availability of a national health technology policy [37] and a unit in the ministry of health responsible for the management of medical devices [37].

The fourth category was on health financing, and the variables were general government expenditure on health as a percentage of total health expenditure, general government expenditure on health as a percentage of total government expenditure, private expenditure on health as a percentage of total expenditure on health, out-of-pocket expenditure on health as a percentage of private expenditure on health, external resources for health as a percentage of total expenditure on health, per capita total expenditure on health, and per capita government expenditure on health [35].

The fifth category was on information and focussed on national health information systems, national health research systems, and research ethics. Data for this category were from a review of published literature.

The sixth category dealt with leadership/governance, and specifically the existence of a national health policy and strategic plan [37],[38].

The seventh category was on essential interventions for maternal health, and specifically antenatal care coverage, births attended by skilled health personnel, family planning services and contraceptive prevalence [35].

The eighth category dealt with immunisation coverage for 1-year-olds with measles, DPT3, HepB3 and Hib3 vaccines [35].

The ninth category was on coverage of health interventions for reversing the spread of HIV/AIDS, and the variables were proportion of the population aged 15¿24 years with comprehensive, correct knowledge on HIV/AIDS, antiretroviral therapy coverage for PMTCT (prevention of mother-to-child-transmission of HIV) among pregnant women infected with HIV, and antiretroviral therapy coverage [35].

The tenth category dealt with coverage of health interventions for malaria control, and the variables were percentage of children aged under 5 years sleeping under insecticide treated nets and availability of antimalarial treatment for children with fever [35].

The eleventh category was on health interventions for the control of tuberculosis, and the variables were the case detection rate for all forms of tuberculosis and the success rate of the smear-positive tuberculosis treatment [35].

The data on human resources for health, health technologies (computed tomography, radiotherapy, and mammography units; and selected generic medicines), health financing, coverage of maternal health services, coverage of immunisation services, and coverage of health services for reversing the spread of HIV/AIDS, malaria and tuberculosis were obtained from the World Health Statistics Report 2014 [35], whilst the data on health facilities were from the WHO Global Health Observatory [36]. The descriptive statistics and correlations were calculated using Stata software [39].

### Study limitations

The analysis reported in this paper is based on data from many secondary sources. Each data source has unique assumptions and data collection and processing methodology. For example, some of the sources are country reports, others are specific data collection efforts, and still others are a mix of these two. The methods used in each source are contained in the references cited in the ¿Data¿ section, with varying levels of detail on the data collection procedures employed. These limitations underscore the need for more investment to strengthen national health information systems and establish national health observatories to facilitate generation of reliable and timely data [40].

### Ethical clearance

Since this study entailed solely analysis of data from the WHO Global Health Observatory and published secondary sources, and since human subjects were not to be involved, it did not require ethical clearance.

## Results and discussion

### Synthesis of health system components

#### Human resources for health

In 2013, globally there were estimated 8 652 107 physicians, 16 689 250 nursing and midwifery personnel, 1 227 822 dentistry personnel, 2 114 282 pharmaceutical personnel, and 169 602 psychiatrists [35]. In spite of the fact that the African Region bears about 25% of the global burden of disease, it had a paltry 1.4% of the physicians, 2.8% of the nursing and midwifery personnel, 1.4% of the dentistry personnel, 1.6% of the pharmaceutical personnel, and 0.7% of the psychiatrists available globally in 2013.

The densities of the health workforce in majority of the countries were extremely low. For example, 53% (19/36) of the countries had fewer than one physician per 10 000 population, 44% (15/34) had fewer than five nursing and midwifery personnel per 10 000 population, 89% (25/28) had fewer than one dentistry personnel per 10 000 population, 85% (28/33) had fewer than one pharmaceutical personnel per 10 000 population, and 89% (39/44) had fewer than 0.05 psychiatrists per 10 000 population. As shown in Table 1, physicians, nursing and midwifery personnel, dentistry personnel, pharmaceutical personnel, and psychiatrists¿ densities were between two and six times lower than global averages [35].

**Table 1:** Health workforce gaps in 2013

**Inputs**	**African region average (A)**	**Global average (B)**	**Gap [C?=?B/A)]**
Physicians per 10 000 population	2.6	14.1	5.4-fold
Nursing and midwifery personnel per 10 000 population	12.0	29.2	2.4-fold
Dentistry personnel per 10 000 population	0.5	2.7	5.4-fold
Pharmaceutical personnel per 10 000 population	0.9	4.3	4.8-fold
Psychiatrists per 10 000 population	<0.05	0.3	6.0-fold

Source: WHO [35].

It is estimated that the African Region has a shortage of at least 817 992 health workers [41]. According to the World Health Report 2006 [41], out of the 57 countries identified as facing a human resources for health (HRH) crisis with health workforce density ratios below 2.3 per 1000 population, 36 were in the African Region. Health workforce shortages in the Region have been attributed to inadequate institutional capacity for HRH management, low levels of national investment in HRH production, slow progress in educational reforms, skewed distribution of health workers, lack of incentives, and ineffective retention strategies.

#### Health facilities

The densities of primary, secondary and tertiary level health facilities in the African Region were lower than those recommended by WHO. For instance, 56% (15/27) of the countries had fewer than 10 health posts per 100 000 population, 85% (28/33) had fewer than 10 health centres per 100 000 population, 82% (28/34) had fewer than one district hospital per 100 000 population, and 100% (27) had fewer than one provincial hospital per 100 000 population. Worse still, 41% of the countries had fewer than 0.1 provincial hospitals and 66% (21/32) fewer than 0.1 tertiary hospitals per 100 000 population [36].

The low densities of primary and secondary level health facilities might partially explain why many countries had low coverage of essential public health interventions and curative services for case management of communicable and non-communicable diseases. Furthermore, the very low densities of tertiary hospitals may undermine African countries¿ capacity to provide specialised referral services, produce physicians and other specialised health workforce cadres, conduct basic and applied research, and utilise available medical innovations.

#### Health technologies

Generally, in the African Region the medical equipment used in the diagnosis of various health problems is grossly inadequate (see Table 2). For example, in 2013, 83% (20/24) of the reporting countries had fewer than one magnetic resonance imaging unit per a million population, 95% (19/20) had fewer than one radiotherapy unit per a million people, 68% (23/34) had fewer than one computed tomography unit per a million population, and 47% (14/30) had fewer than 10 mammography units per a million population. There were 0.1 radiotherapy units per a million population in the Region compared with 1.8 units globally [35],[36]. Thirty-eight percent (16/42) of countries had a health technology policy and 86% (36/42) had a health technology management unit.

**Table 2:** Health technology gaps in 2013

**Medical devices and beds**	**African region average (A)**	**Global average (B)**	**Gap [C?=?B/A)]**
Computed tomography units per million population	0.4	1.9 (at Eastern Mediterranean Region)	4.8-fold
Radiotherapy units per million population	0.1	1.8	18.0-fold
Mammography units per million females aged 50 to 69 years	7.4	20.9	2.8-fold
Hospital beds per 10 000 population (2012)	9	27	3.0-fold
Psychiatric beds per 10 000 population (2010)	0.6	2.5	4.2-fold
Hospitals per 10 000 population	0.8	0.9 (at Eastern Mediterranean Region)	1.1-fold

Source: WHO [35].

Availability of *essential medicines* in public health facilities varied widely, ranging from 17.9% to 87.1%, and in private health facilities from 13.6% to 88% [35]. WHO estimates that in Africa about half of those in need of essential medicines do not have access to them [42], mainly because of cost reasons. The *median consumer price ratio (MPR)* is the ratio of the local price to an international reference price converted into the local currency. An MPR of 1 means that the local price is equivalent to the reference price, and an MPR of 2 that the local price is twice the reference price [35]. The MPR among public health facilities varied from 1.3 to 6.5, meaning that in some cases the local price was almost seven times the international reference price [35]. The MPR among private health facilities ranged from 2.2 to 15.1. In 53% of the countries, the local prices of selected generic medicines in the private health sector were more than four times the international reference prices. MPRs in the private health sector were remarkably higher than those in the public health sector [35]. The main challenges related to essential medicines are associated with their limited access; the circulation of fake, substandard and counterfeit medicines; weak regulatory capacity; non-rational use of medicines; and weak research and innovation capacity to address priority needs [43].

#### Health financing

The data used for this section were for 2011. On average in the African Region general government expenditure on health constituted 48.7% of total expenditure on health, compared with the global level of 57.8%. *G*ov*ernment expenditure on health as a percentage of total government expenditure* was 9.7%, compared with 15.2% globally. By the end of 2011, only six of the 45 countries reporting on this factor had met the Abuja target of allocating at least 15% of total government expenditure to the health sector.

The *private expenditure on health* constituted 51.7% of total expenditure on health compared with 41.1% globally. About 56.5% of the private expenditure on health was from household out-of-pocket spending, compared with the global level of 48.8%, and 31.3% was from private prepaid plans, compared with 37.2% globally. The fact that out-of-pocket payments form over 50% of private spending on health in 38 (83%) of the countries is of great concern given that such a high level of spending on health exposes households to the risk of financial catastrophe and impoverishment. In fact, there is evidence that where out-of-pocket spending is less than 20% of total health expenditure, the risk of catastrophic health expenditure is negligible. Unfortunately, in 2010, out-of-pocket payments as a share of total health expenditure were above 20% in 35 countries, meaning that significant sizes of the population in those countries were exposed to the risk of financial catastrophe and impoverishment.

*External resources for health* made up less than 20% of total health expenditure in 21 (46%) of the countries, 20-40% in 15 countries, and over 40% in 10 countries. The average donor contribution to total health expenditure was 11.8%, compared with 0.4% globally. For 22 countries (45.7%) more than 25% of the expenditure on health was from external resources.

In 2009, the High Level Taskforce on Innovative International Financing for Health Systems estimated that on average US$ 44 per capita???rising to US$ 60 in 2015???would be needed to ensure coverage with a set of essential health services in 49 low-income countries [44]. *Per capita total expenditure on health* was less than US$ 44 in 21 countries, US$ 44 to US$ 60 in four countries and over US$ 60 in 21 countries. Per capita total expenditure on health varied widely, going from US$ 12 to US$ 1051. As shown in Table 3 the average per capita total spending on health was US$ 99, compared with US$ 1007 globally, more than tenfold lower.

**Table 3:** Health expenditure gaps in 2011

**Variables**	**African region average (A)**	**Global average (B)**	**Gap [C?=?B/A)]**
Total expenditure on health as% of gross domestic product	6.2	9.1	1.5-fold
General government expenditure on health as% of total expenditure on health	48.3	58.8	1.2-fold
Private expenditure on health as% of total expenditure on health	51.7	41.1	0.8-fold
General government expenditure on health as% of total government expenditure	9.7	15.2	1.6-fold
External resources for health as% of total expenditure on health	11.8	0.4	0.03-fold
Social security expenditure on health as% of general government expenditure on health	8.0	60.6	7.6-fold
Out-of-pocket expenditure as% of private expenditure on health	56.6	49.7	0.9-fold
Private prepaid plans as% of private expenditure on health	31.7	38.2	1.2-fold
Per capita total expenditure on health at average exchange rate (US$)	99	1007	10.2-fold
Per capita total expenditure on health (PPP Int$)	158	1053	6.7-fold
Per capita government expenditure on health at average exchange rate (US$)	49	613	12.5-fold
Per capita government expenditure on health (PPP Int$)	76	619	8.1-fold

Source: WHO [35].

*Per capita government expenditure on health* was less than US$ 10 in seven countries, US$ 10 to US$ 30 in 21 countries and over US$ 30 in 18 countries. The range was from US$ 6 to US$ 570. The African Region¿s average per capita government expenditure on health was US$ 49, compared with US$ 613 globally. Some 46% of the countries had a per capita government expenditure on health of less than US$ 20.

#### Information

Mbondji and colleagues¿ [20] questionnaire-based survey found that 36% (16/44) of the countries in sub-Saharan Africa had a functional national health research governance mechanism, 20% (9/44) had clear terms of reference and 20% had a functional national health research management forum. Kebede and colleagues¿ [44] questionnaire survey of 847 health research institutions revealed that 49% of these institutions had computer laboratories, 50% had network computers, 38% had information technology support, and 67% had a library.

The descriptive analysis of data sources undertaken by Mbondji and colleagues [18] revealed that in almost all countries in the Region there was heavy reliance on household surveys for indicators. Few countries had civil registration systems that permitted adequate and regular tracking of mortality and cause of death data. The health management information systems generated considerable data but these were used rarely because of concerns about bias, quality and timeliness. About 93% (43/46) of the countries in the Region had introduced an integrated disease surveillance and response system.

In Mbondji and colleagues¿ [20] questionnaire survey, 75% (33/44) of the countries reported to have functional ethical review committees. About 57% of the 847 health research institutions surveyed by Zielinski and colleagues [21] responded to the survey, with 51% of these reporting to have policies on research ethics, 58% to have written policies requiring researchers to obtain informed consent of research participants, 34% to have established ethics review committees, 42% to require that studies go through ethics review committees, 46% to have linkages with national or regional ethics organisations, 53% to have adopted standard operating procedures, and 36% to provide some type of ethics training for staff [36].

#### Leadership and governance

Although no readily available indicators exist for the leadership and governance component of health systems, it is a crucial element for ensuring that appropriate strategic policy frameworks exist for the pursuit of the UHC vision and are combined with effective and accountable building of partnerships. In this study we used three indicators for this component: existence of a valid national health policy and strategic plan. About 87% (41/47) of the countries had a valid national health policy and strategic plan [37].

### Progress towards universal coverage of MDG-related interventions

#### MDG 5: Maternal health

Generally, as shown in Table 4, the regional coverage of maternal health interventions coverage was lower than the global averages. In 2013, the average *antenatal care (ANC)* coverage involving at least one visit with a health worker was 75% in the African Region, compared with 81% globally, and coverage involving at least four visits with a health worker was 47%, compared with 56% globally. Coverage among the countries ranged from 15% to 97%. Out of the 39 countries reporting, ANC coverage involving at least four visits was lower than 40% in nine countries, between 40% and 60% in 14 countries and over 60% in 16 countries [35]. In 2013, only 48% of the *births were attended by skilled health personnel,* compared with 72% globally [35]. Among the countries, this proportion varied from 10% to 100% [35].

**Table 4:** Maternal health services gaps in 2013

**Services**	**African region average (A)**	**Global average (B)**	**Gap [C?=?B/A)]**
Unmet need for family planning in 2012 (%)	25	12	0.5-fold
Contraceptive prevalence in 2012 (%)	27	63	2.3-fold
Antenatal care coverage: at least one visit in 2013 (%)	75	81	1.1-fold
Antenatal care coverage: at least four visits in 2013 (%)	47	56	1.2-fold
Births attended by skilled health personnel in 2013 (%)	48	72	1.5-fold
Births by caesarean section in 2012 (%)	4	16	4.0-fold
Postnatal care visits within two days of childbirth in 2012 (%)	41	48	1.2-fold

Source: WHO [35].

**Figure 2:**
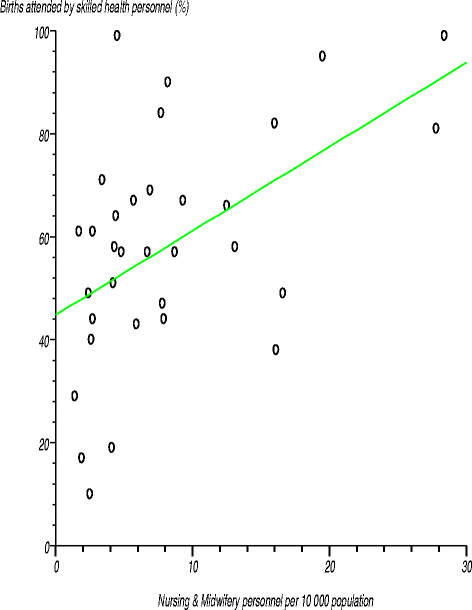
Relationship between percentage of births attended by skilled health personnel and nursing and midwifery personnel per 10 000 population.

We tested the null hypothesis that no relationship existed between the percentage of births attended by skilled health personnel and number of health facilities, personnel numbers in various health workforce cadres, health spending, and availability of medical devices. We found that the correlation coefficients were significantly different from zero at the 97.5% confidence level for physicians per 10 000 population, nursing and midwifery personnel per 10 000, general government expenditure on health as a percentage of total health expenditure, private expenditure on health as a percentage of total health expenditure, per capita total expenditure on health, and radiotherapy units per a million population. Figure 2 shows a positive relationship between percentage of births attended by skilled health personnel and nursing and midwifery personnel per 10 000 population. The correlation coefficient (r) was 0.509946 (r^2^?=?0.260045), computed t-statistic with 31 degrees of freedom was 3.300676, two-sided P was 0.0024 and critical t-statistic from the statistical tables was 1.960 at the 97.5% confidence level. Since the computed t-statistic was higher than the critical t value, we rejected the null hypothesis (H_0_) that there is no relationship between the two variables. We concluded that the correlation between the percentage of births attended by skilled health personnel and nursing and midwifery personnel per 10 000 population was significantly different from zero at the 97.5% confidence level.

**Figure 3:**
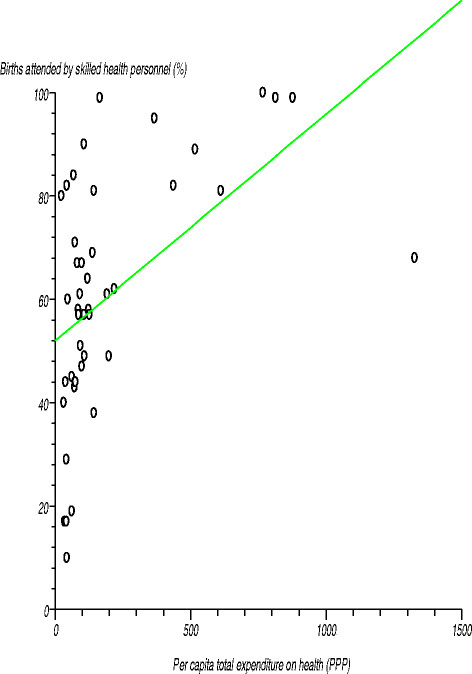
Relationship between percentage of births attended by skilled health personnel and per capita total expenditure on health.

Figure 3 depicts the relationship between percentage of births attended by skilled health personnel and per capita total expenditure on health. We tested the null hypothesis that there was no relationship between these two variables. The estimated correlation coefficient (r) was 0.51424 (r^2^?=?0.264442), computed t-statistic at 42 degrees of freedom was 3.885813, two-sided P was 0.0004 and critical t-statistic was 1.960 at the 97.5% confidence level. Since the computed t-statistic was greater than the critical t-statistic, we rejected the null hypothesis and concluded that the correlation coefficient was significantly different from zero. There was a positive relationship between the percentage of births attended by skilled health personnel and per capita total expenditure on health.

The correlation coefficient for the relationship between the percentage of births attended by skilled health personnel and general government expenditure on health as a percentage of total health expenditure was positive and significantly different from zero. On the other hand, the correlation coefficient for private expenditure on health as a percentage of total health expenditure was significantly different from zero but negatively related to the percentage of births attended by skilled health personnel.

**Figure 4:**
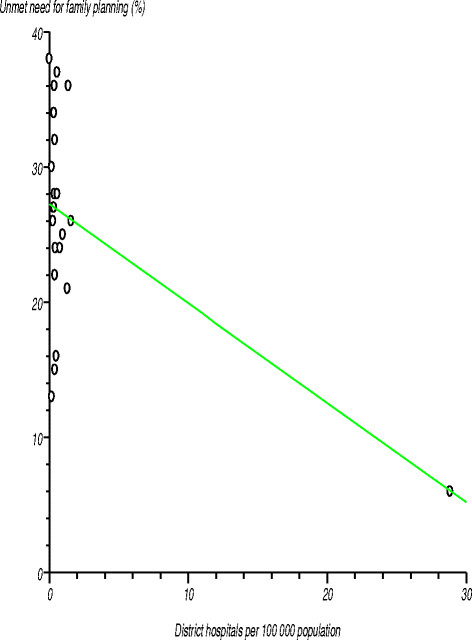
Relationship between unmet need for family planning and number of district hospitals per 100 000 population in the African Region.

*Unmet need for family planning services* in the African Region stands at 25%, compared with the global average of 12%. The levels varied widely among the countries, ranging from 6% to 38% [35]. Figure 4 depicts the relationship between unmet need for family planning services and the number of district hospitals per 100 000 population. A test was conducted on the null hypothesis that there was no relationship between these two variables, i.e. the correlation coefficient was not significantly different from zero. The estimated correlation coefficient (r) was ?0.539349 (r^2^?=?0.290898), computed t-statistic with 21 degrees of freedom was ?2.935117, two-sided P was 0.0079 and critical t-statistic from statistical tables was 1.721 at the 95% confidence level. Since the computed t-statistic was greater than the critical t-statistic, we rejected the null hypothesis and concluded that the correlation coefficient was significantly different from zero; that is, there was a negative relationship between unmet need for family planning services and the number of district hospitals per 100 000 population. The coefficients for other levels of health facilities, various categories of human resources, per capita total expenditure on health, and medical devices were not significantly different from zero.

**Figure 5:**
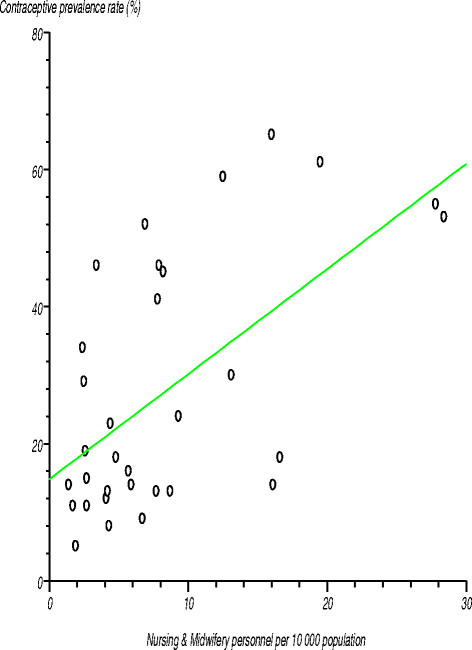
Relationship between contraceptive prevalence rate and nursing and midwifery personnel per 10 000 population.

The Region¿s *contraceptive prevalence rate* of 27% was much lower than the global average of 63%. Contraceptive prevalence among the countries varied from 4% to 65% [35]. Figure 5 shows the relationship between contraceptive prevalence rate and nursing and midwifery personnel per 10 000 population. We tested the null hypothesis that there was no relationship between these two variables and, thus, that the correlation coefficient was not significantly different from zero. The estimated correlation coefficient (r) was 0.589924 (r^2^?=?0.34801), computed t-statistic at 30 degrees of freedom was 4.001619, two-sided P was 0.0004 and critical t-statistic was 1.960 at the 97.5% confidence level. Since the computed t-statistic was greater than the critical t-statistic, we rejected the null hypothesis and concluded that the correlation coefficient was significantly different from zero. There was a positive relationship between contraceptive prevalence rate and the number of nursing and midwifery personnel per 10 000 population. The correlation coefficient for the number of physicians per 10 000 population was positive and significantly different from zero, signifying the existence of a relationship between this variable and contraceptive prevalence rate.

**Figure 6:**
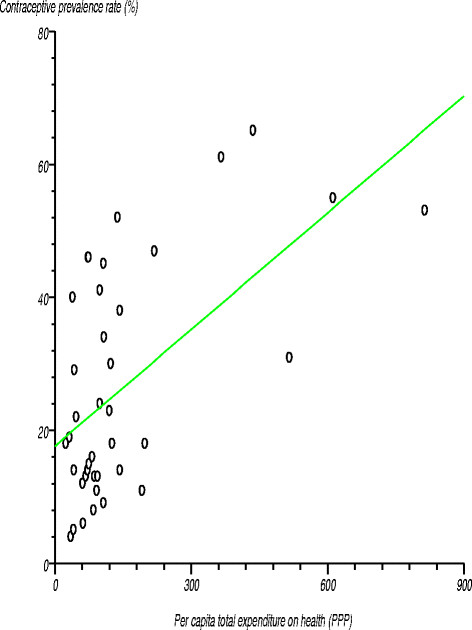
Relationship between contraceptive prevalence rate and per capita total expenditure on health.

Figure 6 shows the relationship between contraceptive prevalence rate and per capita total expenditure on health. We tested the null hypothesis that there was no relationship between these two variables and, thus, the correlation coefficient was not significantly different from zero. The estimated correlation coefficient (r) was 0.578638 (r^2^?=?0.334822), computed t-statistic at 37 degrees of freedom was 4.315581, two-sided P was 0.0001 and critical t-statistic was 1.960 at the 97.5% confidence level. Since the computed t-statistic was greater than the critical t-statistic, we rejected the null hypothesis and concluded that the correlation coefficient was significantly different from zero. There was a positive relationship between contraceptive prevalence rate and per capita total expenditure on health.

#### MDG 4: Routine immunisation coverage

**Figure 7:**
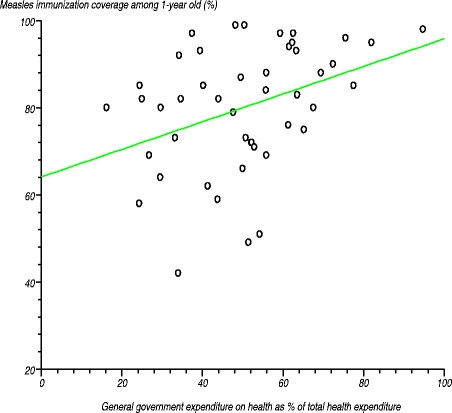
Relationship between measles immunisation coverage among 1-year-olds and general government expenditure on health as a percentage of total health expenditure.

As portrayed in Table 5, African Region¿s average *measles immunisation coverage* among 1-year-olds was 73%, compared with the global average of 84% [35]. Measles immunisation coverage varied among the countries, ranging from 42% to 99%. Figure 7 shows the relationship between measles immunisation coverage among 1-year-olds and general government expenditure on health as a percentage of total health expenditure. We tested the null hypothesis that there was no relationship between these two variables. The estimated correlation coefficient (r) was 0.376021 (r^2^?=?0.141392), computed t-statistic at 44 degrees of freedom was 2.691791, two-sided P was 0.01 and critical t-statistic was 1.960 at the 97.5% confidence level. Since the computed t-statistic was greater than the critical t-statistic from the statistical tables, we rejected the null hypothesis at the 95% confidence level. We concluded that the correlation coefficient was significantly different from zero, meaning that there was a positive relationship between measles immunisation coverage among 1-year-olds and general government expenditure on health as a percentage of total health expenditure. This makes even more sense when we recall that in national health accounts estimates general government expenditure on health also includes funding from partners [45].

**Table 5:** MDG 4 health interventions gaps in 2012

**Interventions**	**African region average (A)**	**Global average (B)**	**Gap [C?=?B/A)]**
Measles (MCV) immunization coverage among 1-year-olds (%)	73	84	1.2-fold
BCG immunization coverage among 1-year-olds (%)	80	89	1.1-fold
DPT3 immunization coverage among 1-year-olds (%)	72	83	1.2-fold
Hib3 immunization coverage among 1-year-olds (%)	65	45	0.7-fold
HepB3 immunization coverage among 1-year-olds (%)	72	79	1.1-fold

Sources: WHO [35].

We also tested the null hypothesis that there was no relationship between measles immunisation coverage among 1-year-olds and private expenditure on health as a percentage of total health expenditure. The estimated correlation coefficient (r) was 0.376021 (r^2^?=?0.141392), computed t-statistic at 44 degrees of freedom was 2.691791 and two-sided P equalled 0.01. As the computed t-statistic of 2.691791 was greater than the critical t value of 1.960 from the statistical tables, we rejected the null hypothesis at the 97.5% confidence level. We concluded that the correlation coefficient was significantly different from zero, meaning that there was an inverse relationship between measles immunisation coverage among 1-year-olds and private expenditure on health as a percentage of total health expenditure. This implies that private health spending, which largely constitutes of direct household out-of-pocket payments in the Region, adversely affects access to immunization services, and hence, immunization coverage.

*Diphtheria tetanus toxoid and pertussis (DTP3) vaccine coverage* among 1-year-olds for the African Region was 72%, compared with the global average of 83%. Coverage among the countries varied from 33% to 99% [35]. *HepB3 immunisation coverage* among 1-year-olds was 72%, compared with the global average of 79%. Coverage among the countries ranged from 41% to 99% [35]. The regional average of 65% for *haemophilus influenzae type B vaccine (Hib3)* coverage among 1-year-olds was higher than the global average of 45%. Coverage varied widely among the countries, ranging from 10% to 99% [35].

#### MDG 6: Coverage of health interventions for combating HIV/AIDS, malaria and tuberculosis

In 2010, in the African Region 35% of the male and 29% of the female population aged 15¿24 years had *comprehensive, correct knowledge on HIV/AIDS*; that is, they could correctly identify the ways of preventing sexual transmission of HIV and reject misconceptions about HIV transmission. The ranges among the countries for this variable were 16% to 55% for the male population and 8% to 59% for the female population [35].

**Figure 8:**
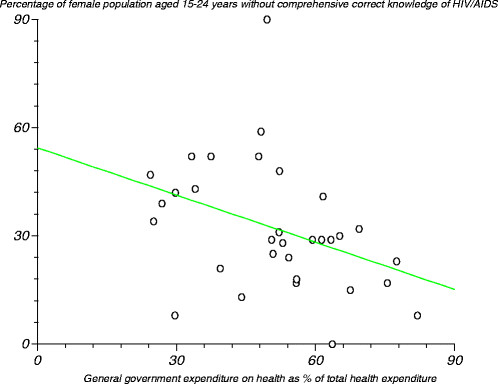
Relationship between percentage of female population aged 15¿24 years without comprehensive, correct knowledge on HIV/AIDS and general government expenditure on health as a percentage of total health expenditure.

Figure 8 depicts the relationship between the percentage of female population aged 15¿24 years without comprehensive, correct knowledge on HIV/AIDS and general government expenditure on health as a percentage of total health expenditure. We tested the null hypothesis that there was no relationship between these two variables. The estimated correlation coefficient (r) was ?0.380545 (r^2^?=?0.144815), computed t-statistic at 30 degrees of freedom equalled ?2.253912, two-sided P was 0.0317 and critical t-statistic was 1.960 at the 97.5% level of significance. Since the computed t-statistic was greater than the critical t-statistic from the statistical tables, we concluded that the correlation coefficient was significantly different from zero. That means that there was a relationship between the percentage of the female population aged 15¿24 years without comprehensive, correct knowledge on HIV/AIDS and general government expenditure on health as a percentage of total health expenditure. This means that as government spending on health increases, health promotion (including health education) programmes would be expected to receive more funding, and hence health illiteracy would be expected to diminish. However, the correlation coefficients for various densities of health facilities, numbers in various health workforce cadres and medical devices were not significantly different from zero.

**Figure 9:**
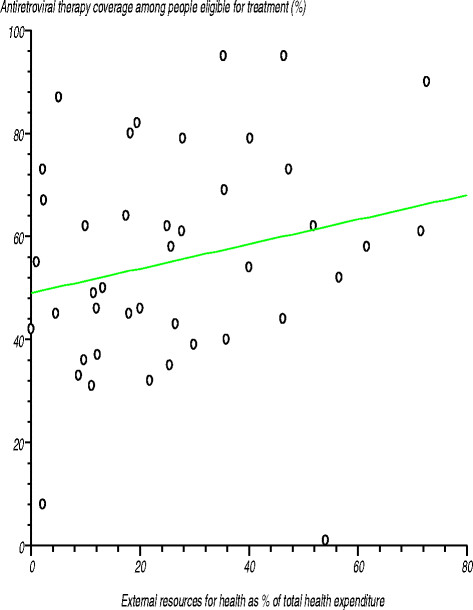
Relationship between antiretroviral therapy coverage among people eligible for that treatment and the level of external resources for health as a percentage of total expenditure on health.

The African Region¿s 2012 average *antiretroviral therapy* coverage among people eligible for that treatment was 63%, compared with the global average of 61% [35]. For the 41 countries reporting on this, antiretroviral therapy coverage varied widely, ranging from 1% to 95% [35]. We tested the null hypothesis that there was no relationship between antiretroviral therapy coverage and densities of the various levels of health facilities and health workforce cadres, health spending level and medical devices. In all cases the correlation coefficient was not significantly different from zero. Figure 9 serves as an example and shows the relationship between antiretroviral therapy coverage among people eligible for that treatment and the level of external resources for health as a percentage of total expenditure on health. The null hypothesis was that there was no relationship between the two variables. The estimated correlation coefficient (r) was 0.29535 (r^2^?= 0.087231), computed t-statistic at 40 degrees of freedom was 1.431704, two-sided P was 0.16 and critical t-statistic from the statistical tables was 1.645 at the 95% level of confidence. Since the computed t-statistic was lower than the critical t-statistic we concluded that the correlation coefficient was not significantly different from zero.

In the African Region in 2012, 64% of pregnant women with HIV received antiretrovirals for *prevention of mother-to-child transmission* of HIV (PMTCT), compared with the global average of 62%. Coverage of antiretrovirals for PMTCT varied widely among the countries, ranging from 13% to over 95% [35].

#### Coverage of health interventions for reversing malaria burden

**Figure 10:**
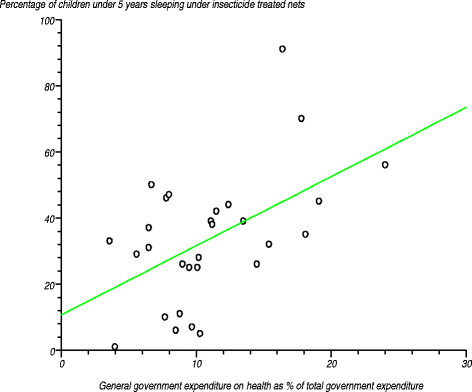
Relationship between percentage of children under 5 years sleeping under insecticide treated nets and general government expenditure on health as a percentage of total government expenditure.

As depicted in Table 6, on average 25% of children aged under 5 years were sleeping under *insecticide treated nets* in the African Region. Among the 29 countries reporting on this item, coverage varied from 1% to 91% [35]. Figure 10 depicts the relationship between percentage of children under 5 years sleeping under insecticide treated nets and general government expenditure on health as a percentage of total government expenditure. We tested the null hypothesis that the correlation coefficient was not significantly different from zero. The estimated correlation coefficient (r) was 0.509 (r^2^?= 0.259081), computed t-statistic at 27 degrees of freedom was 3.07266, two-sided P was 0.0048 and critical t-statistic was 2.052 at the 97.5% confidence level. Since the computed t-statistic was greater than the critical t-statistic, we rejected the null hypothesis and concluded that the correlation coefficient was significantly different from zero. This means that there was a positive relationship between the percentage of children under 5 years sleeping under insecticide treated nets and general government expenditure on health as a percentage of total government expenditure.

**Table 6:** MDG 6 health interventions gaps in 2012

**Interventions**	**African region average (A)**	**Global average (B)**	**Gap [C?=?B/A)]**
Children aged <5 years sleeping under insecticide-treated nets (%)	25	30 (in low income countries)	1.2-fold
Children aged <5 years with fever who received treatment with any antimalarial (%)	-	-	-
Antiretroviral therapy coverage among people eligible for treatment (%)	63	61	0.96-fold
Pregnant women with HIV receiving antiretrovirals to prevent mother-to-child-transmission (MTCT)	64	62	0.97-fold
Case-detection rate for all forms of tuberculosis (%)	59	67	1.1-fold
Treatment-success rate for smear-positive tuberculosis in 2011 (%)	82	87	1.1-fold

Source: WHO [35].

**Figure 11:**
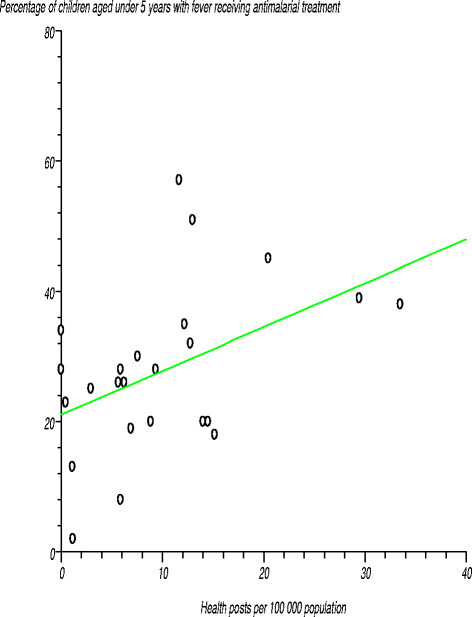
Relationship between percentage of children aged under 5 years with fever receiving antimalarial treatment and number of health posts per 100 000 population.

Among the 36 countries reporting on coverage of *antimalarial treatment* for fever, the levels ranged from 2% to 65% [35]. Figure 11 shows the relationship between percentage of children aged under 5 years with fever receiving antimalarial treatment and number of health posts per 100 000 population. We tested the null hypothesis that there was no relationship between the two variables. The estimated correlation coefficient (r) was 0.458264 (r^2^?=?0.210006), computed t-statistic at 22 degrees of freedom was 2.418329 and two-sided P equalled 0.0243. Since the computed t-statistic was greater than the critical t-statistic value of 2.074 from the statistical tables, we rejected the null hypothesis at the 97.5% confidence level. We concluded that the correlation coefficient was significantly different from zero, implying that there was a positive relationship between the percentage of children aged under 5 years with fever receiving antimalarial treatment and number of health posts per 100 000 population. The correlation coefficients for the densities of health facilities in the other levels of the system, i.e. district, provincial and tertiary hospitals, were not significantly different from zero.

#### Coverage of health interventions for tuberculosis control

**Figure 12:**
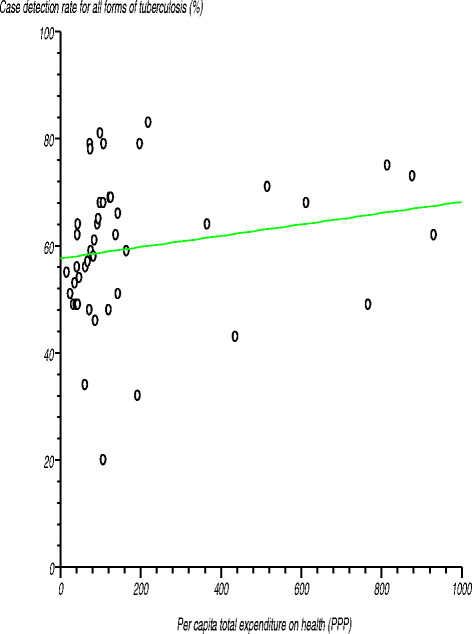
Relationship between case detection rate for all forms of tuberculosis and per capita total expenditure on health.

In 2012, the African Region had the lowest average *case detection rate for all forms of tuberculosis,* at 59%, compared with the global average of 67%. This rate varied widely among the countries, ranging from 20% to 83% [35]. We found no relationship between case detection rate for all forms of tuberculosis and densities of various levels of health facilities, cadres of the health workforce, medical devices or spending on health. Figure 12 shows the relationship between case detection rate for all forms of tuberculosis and per capita total expenditure on health. The estimated correlation coefficient (r) was 0.188845 (r^2^?= 0.035663), computed t-statistic at 43 degrees of freedom was 1.261032, two-sided P was 0.2141 and critical t-statistic was 1.645 at the 95% confidence level. Since the computed t-statistic was lower than the critical t-statistic, we accepted the null hypothesis that the correlation coefficient was not significantly different from zero.

**Figure 13:**
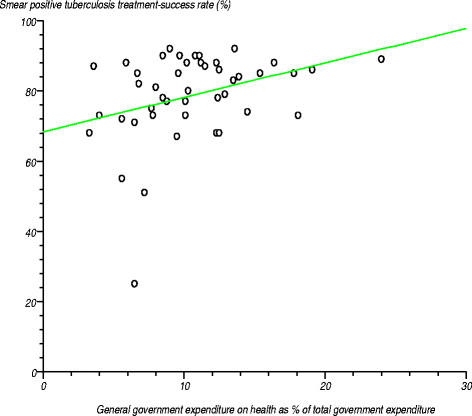
Relationship between smear positive tuberculosis treatment success rate and general government expenditure on health as a percentage of total government expenditure.

In 2011, the African Region had a success rate of 82% for *smear-positive tuberculosis treatment* compared with the global average of 87%. There was wide variation in this rate among the countries, ranging from 25% to 92% [35]. Figure 13 portrays the relationship between the smear positive tuberculosis treatment success rate and general government expenditure on health as a percentage of total government expenditure. We tested the null hypothesis that the correlation coefficient was not significantly different from zero. The estimated correlation coefficient (r) was 0.338786 (r^2^?=?0.114776), computed t-statistic at 43 degrees of freedom equalled 2.361204, two-sided P was 0.0228 and critical t-static from the statistical tables was 1.960 at the 97.5% confidence level. The computed t-statistic was greater than the critical t-statistic, so we rejected the null hypothesis and concluded that the correlation coefficient was significantly different from zero.

### Major thrusts towards universal health coverage

The gaps in human resources, facilities, technologies and financing for health in many instances are correlated with gaps in coverage of public health services. Closing these gaps will call for increased health financing and risk pooling mechanisms that shield poor people from catastrophic health expenditures, and scaling up of interventions and access to quality health care throughout the life-cycle.

According to the World Health Report 2010, three problems restrict countries¿ movement towards achieving universal health coverage: scarcity of resources, overreliance on out-of-pocket payments and inefficient and inequitable use of resources [46]. What can countries do to successfully move towards UHC?

We propose four thrusts for attainment of the universal health coverage goal: strengthening public health infrastructure capacity, raising sufficient resources to strengthen health systems, promoting efficiency in national health services to optimise resource use and maximise results, and removing financial risks and barriers to care and service access.

#### Strengthening public health infrastructure capacity

We define health infrastructure as the essential resources that are required for the delivery of health care. Infrastructure encompasses human resources, technologies and facilities [47].

The road map for expanding human resources for health for improved health service delivery in the African Region during 2012¿2025 [48],[49] proposes six thrusts to countries: strengthening leadership and governance; strengthening regulatory capacity; scaling up education and training; optimising the deployment, retention and performance of available personnel; improving the generation of information to support evidence-based decision-making; and strengthening partnership and dialogue. In order for health workers to apply their knowledge and skills to solve specific health problems they need essential health technologies developed for that purpose. Acquisition of health technology should take cognisance of the available related infrastructure components. Each country ought to have a national health technology policy as an integral part of the overall national health policy, legislation or plan [50]-[52]. Effective implementation of that policy presupposes existence of skilled staff involved in selection, planning, procurement, management, maintenance and utilisation of health technologies.

Health posts and centres and district hospitals provide health services in a health district with a population of 100 000 and 300 000. Walley and colleagues [32] posit that for district health systems to work effectively, countries need to adopt four strategies: definition of the roles of health facilities, integration of health care, building and strengthening of the referral system, and decentralisation of management. District health systems form the operational level of primary care, and that role should be articulated. The relationship of district health systems with health facilities at secondary and tertiary levels, as well as the role of hospitals and public health programmes within the national health services, needs to be clearly defined.

#### Raising sufficient resources to strengthen health systems

We believe it is possible to increase domestic resources through improvement of the efficiency of tax revenue collection; prioritisation of government budgets to meet the 15% requirement defined by the Abuja commitment [46]; innovative financing, such as taxing tobacco, alcohol, air tickets, foreign exchange transactions, mobile phone use and diaspora remittances [53]; and leveraging the private sector [54]. Predictable, aligned, harmonised and increased development assistance for health is critically necessary, especially for low income countries [55],[56].

#### Promoting efficiency in national health services to optimise resource use and maximise results

Efficiency is essential in allocation of health resources and provision of national health services to optimise resource use and maximise results. The World Health Report 2010 on health systems financing estimated that about 20-40% of the resources spent on health are wasted [46]. Such resources could have been directed towards the pursuit of universal health care coverage. A number of tools exist for addressing health resource gaps in the African Region, including the Ouagadougou Declaration on Primary Health Care and Health Systems in Africa [10] and its implementation framework [11], the Algiers Declaration on Research for Health [57] and its implementation framework [58], the Addis Ababa Declaration on Community Health [59] and the Libreville Declaration on Health and Environment [60].

#### Removing financial risks and barriers to care and service access

Compulsory prepayment into a fund for health services before their need arises is recommended as a way of removing financial risks and barriers to access of health services. Where multiple funds exist, there will be need for cross-subsidisation. Prepayment arrangements can be organised through general taxation or compulsory contributions for health insurance or both. Since approximately 51.5% of the African Region¿s population lives on less than Int$ 1 per day [35], the poor will need assistance through either direct access to government-financed health services or subsidies for their health insurance premiums.

Since 2005, we have seen an increase in the number of African countries pursuing the goal of providing universal health coverage, including Benin, Burkina Faso, Congo, Democratic Republic of Congo, Ghana, Gabon, Lesotho, Kenya, Nigeria, Rwanda, South Africa, Swaziland, Uganda and Zambia. It is critically important that Africa draw lessons from countries around the world that have made significant strides in providing universal health coverage through taxation or social health insurance or a mix of the two.

The literature points to a number of factors that have facilitated countries to progress towards universal health coverage: a high per capita income that strengthened the capacity of businesses and citizens to prepay for health care; a large formal sector that enhanced the capacity to contribute and boosted collection of contributions; urbanisation and communication that facilitated the delivery of services; a skilled labour force, including in health services, to deliver the health care benefit packages and manage health financing schemes; solidarity of the society that facilitated cross-subsidisation from the rich to the poor and from the healthy to the ill; strong government stewardship capacity to launch, guide and sustain a process of compulsory prepayment; and availability of public and private health services of ¿acceptable¿ quality [6].

The majority of African countries lack an enabling environment and are characterised by relatively low per capita gross domestic product; large informal agricultural and small commerce/business sectors that are difficult to tax; limited volumes of taxable imports; insufficiently skilled labour force, including a crisis in human resources for health; and structural and institutional weaknesses that might compromise the efficiency of compulsory prepayment policies and mechanisms.

In spite of these challenges, several countries have introduced social health insurance schemes for their populations. So far, the two well-documented success stories are Ghana and Rwanda, which are implementing a mixed financing health insurance system, with Ghana¿s scheme being fundamentally tax based [61] while Rwanda¿s is essentially community based [62].

## Conclusion

This study highlights the current situation of the health system components in the African Region and the way they affect the coverage of health MDGs. It also proposes four major thrusts which could sustain the way towards universal health coverage.

The analysis revealed the existence of substantial deficits in health systems¿ components and access to health care. For example, the African Region¿s average densities of physicians, nursing and midwifery personnel, dentistry personnel, pharmaceutical personnel, and psychiatrists of 2.6, 12, 0.5, 0.9 and 0.05 per 10 000 population were 5.4-fold, 2.4-fold, 5.4-fold, 4.8-fold and 6-fold lower than global averages.

There were gaps also in densities of health facilities in the Region. For instance, 56% of the countries reporting had fewer than 11 health posts per 100 000 population, 88% had fewer than 11 health centres per 100 000 population, 82% had fewer than one district hospital per 100 000, 74% had fewer than 0.20 provincial hospitals per 100 000 population, and 79% had fewer than 0.20 tertiary hospitals per 100 000 population.

For health technologies, 83% of the reporting countries had fewer than one MRI per one million people, 68% had fewer than one computed tomography unit per one million people, 95% had fewer than one radiotherapy unit per a million population, and 47% had fewer than 10 mammography units per a million females aged 50 and 69 years.

In terms of health financing, household out-of-pocket spending on health care made up over 20% of total health expenditure for 34 (76%) of African Region¿s countries, which implies that significant portions of populations in those countries are exposed to a high risk of financial catastrophe. Forty-six percent of the countries had not adopted the recommendation of the International Taskforce on Innovative Financing to spend at least US$ 44 per person per year on health.

Some of the gaps in health system components were found to be correlated to coverage gaps in health MDGs-related services. For example, with regard to the percentage of births attended by skilled health personnel, the correlation coefficients for physicians per 10 000 population, nursing and midwifery personnel per 10 000, general government expenditure on health as a percentage of total health expenditure, private expenditure on health as a percentage of total health expenditure, per capita total expenditure on health, and radiotherapy units per a million population were significantly different from zero at the 97.5% confidence level.

The umbrella post-2015 health goal set at the global level to achieve universal health coverage will be achieved only if two clear global targets are met [63]:

 That by 2030, at least 80% of the poorest 40% of the population have coverage to ensure access to essential health services, and

 That by 2030 everyone ¿ and that means 100% of the population ¿ have coverage protecting them from financial risk so that no one is pushed into poverty or kept in poverty for having spent money to meet health needs.

In January 2014, the African Union adopted a common position on the post-2015 development agenda that recognises universal health coverage as the post-2015 health goal for the continent [64]. The April 2014 Luanda Commitment on Universal Health Coverage in Africa reignited the flame to work towards the achievement of goals defined in existing regional declarations and strategies on health systems strengthening, when all 54 African Union Member States, 47 of which are WHO African Region members, pledged to put the necessary structures and processes in place by 2025 for the attainment of universal health coverage [65]. It is also essential that right now all Member States in the Region look beyond 2015 and plan for future health-related needs based on current evidence, and develop costed road maps for attaining universal health coverage through strengthened health systems¿ that operate in synergy with other government sectors in charge of social and economic determinants of health.

## Competing interests

The authors declare that they have no competing interests.

## Authors¿ contributions

LGS and JMK participated in the design, analysis and writing of the manuscript. Both authors read and approved the final manuscript.
